# The Magpie Trial: a randomised trial comparing magnesium sulphate with placebo for pre-eclampsia. Outcome for women at 2 years

**DOI:** 10.1111/j.1471-0528.2006.01166.x

**Published:** 2006-12-12

**Authors:** Magpie Trial Follow-Up Study Collaborative Group

**Keywords:** Longterm follow-up, magnesium sulphate, pre-eclampsia, randomised trial

## Abstract

**Objective:**

The aim of this study was to assess long-term effects for women following the use of magnesium sulphate for pre-eclampsia.

**Design:**

Assessment at 2–3 years after delivery for women recruited to the Magpie Trial (recruitment in 1998–2001, ISRCTN 86938761), which compared magnesium sulphate with placebo for pre-eclampsia.

**Setting:**

Follow up after discharge from hospital at 125 centres in 19 countries across five continents.

**Population:**

A total of 7927 women were randomised at the follow-up centres. Of these women, 2544 were not included for logistic reasons and 601 excluded (109 at a centre where <20% of women were contacted, 466 discharged without a surviving child and 26 opted out). Therefore, 4782 women were selected for follow-up, of whom 3375 (71%) were traced.

**Methods:**

Questionnaire assessment was administered largely by post or in a dedicated clinic. Interview assessment of selected women was performed.

**Main outcome measures:**

Death or serious morbidity potentially related to pre-eclampsia at follow up, other morbidity and use of health service resources.

**Results:**

Median time from delivery to follow up was 26 months (interquartile range 19–36). Fifty-eight of 1650 (3.5%) women allocated magnesium sulphate died or had serious morbidity potentially related to pre-eclampsia compared with 72 of 1725 (4.2%) women allocated placebo (relative risk 0.84, 95% CI 0.60–1.18).

**Conclusions:**

The reduction in the risk of eclampsia following prophylaxis with magnesium sulphate was not associated with an excess of death or disability for the women after 2 years.

## Introduction

Pre-eclampsia complicates 2–8% of pregnancies.^1^ Although outcome is often good, pre-eclampsia is a major cause of maternal and perinatal morbidity and mortality.^2^–^5^ Whether pre-eclampsia has longer term consequences for the health of both the woman and her child is unclear, although hypertension during pregnancy does seem to be associated with an increase in cardiovascular mortality for the woman in her later life.^6^,^7^

The Magpie Trial,^8^ a large international study, is a randomised comparison of magnesium sulphate with placebo for women with pre-eclampisa. This trial showed that magnesium sulphate halves the relative risk (RR) of eclampsia, without appearing to have substantive harmful effects on either the mother or the baby in the short term.^8^,^9^ To date, there has been no reliable assessment of whether^8^ magnesium sulphate influences long-term outcome following pregnancy complicated by pre-eclampsia.^10^,^11^ We therefore contacted women recruited to the Magpie Trial when their children were 18 months or older. The main objective was to determine whether magnesium sulphate affects the child’s chance of surviving without neurosensory disability, and these data are reported elsewhere.^12^ Secondary objectives, which are reported in this article, were to determine the effects of magnesium sulphate on longer term outcome for women to assess whether there are unexpected adverse events and to assess prognosis for this international cohort of women with pre-eclampsia.

## Methods

Between 1998 and 2001, 10 141 women were recruited to the Magpie Trial at 175 hospitals in 33 countries.^8^ The women were eligible for the trial if they had pre-eclampsia (blood pressure ≥140/90 mmHg and 1+ proteinuria) during pregnancy, if in labour or up to 24 hours postpartum and if there was uncertainty about whether to use magnesium sulphate. They were randomly allocated to receive either magnesium sulphate heptahydrate 50% solution or placebo, given as a loading dose plus 24 hours maintenance therapy.^11^ Each hospital chose whether the maintenance regimen would be intravenous or intramuscular for the duration of the trial. For the active arm, the loading dose was 4 g magnesium sulphate. For the intravenous maintenance regimen, this was followed by an intravenous infusion of 1 g magnesium sulphate per hour. For the intramuscular maintenance regimen, the intravenous loading dose was combined with 10 g magnesium sulphate intramuscularly, followed by 5 g magnesium sulphate intramuscularly every 4 hours.

**Figure 1:**
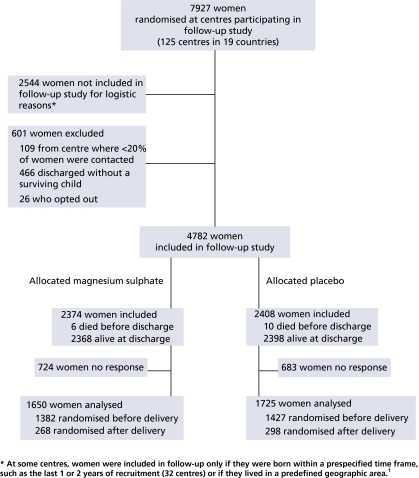
Consort flow for women included in the follow-up.

Of the 10 141 women recruited to the trial, 10 110 were included in the analysis of outcome at discharge from the hospital.^8^ A further 2295 women were never eligible for follow up: 98 for whom there were no data about delivery and outcome at discharge from the hospital and 2197 at 50 centres, predominantly in developing countries, where follow-up was not thought possible. The main reasons for centres not participating in follow up were factors such as mobility of the local population and inaccurate or no contact details provided at discharge from the hospital or that there was no local coordinator, often because the coordinator for the main trial had left. The flow chart of the women who ultimately participated in this follow-up study is shown in Figure 1. Every individual involved in tracing and assessment remained blind to the allocated treatment. The protocol for the follow-up study, which includes examples of the questionnaires, is published elsewhere,^13^ as are narrative accounts from the collaborators.^14^

### Ethics approval and consent

All the hospitals secured local research ethics committee approval before starting recruitment to the main trial. Women were informed that they might be contacted for follow up prior to giving consent for recruitment. Therefore, some centres did not require the follow-up study be resubmitted to an ethics committee. Others required a new submission or considered the follow-up protocol an amendment to the original trial protocol. After discussion within the collaborative group, it was agreed that permission to contact women discharged from the hospital without a surviving child would not be requested. This was a pragmatic decision designed to ensure that our primary objective of assessing outcome for the children could be achieved. It was based on the widespread perception that such permission would be refused and that requesting it might either substantially delay ethics approval or lead to rejection of the study.

### How women were assessed

Women were asked to complete a brief questionnaire asking about their health and wellbeing.^13^ Apart from asking about high blood pressure and use of medicines for the same, all the questions were open ended, with the women asked to record in their own words their health problems and reasons for attending the clinic and/or the hospital. Questionnaires were available in English and Spanish. They were sent by post, administered in clinic or during a home visit or completed over the telephone. If the family could not be contacted, then the information about whether the women and the children were ‘alive and well’ was collected, whenever possible, from neighbours, relatives or outpatient records. If the child was invited for a paediatric assessment,^12^ this opportunity was taken to measure the woman’s blood pressure and to ask her about possible hypertension.

In the UK, an additional questionnaire was sent to the woman’s GP about 18 months after delivery, and the date and cause of the death were provided by the Office for National Statistics.

### Data coding and review

Information collected as free text was coded to reflect the underlying condition or group of conditions. Two obstetricians (Jack Moodley and Edgardo Abalos) reviewed the data for women who died and a third (Lelia Duley) reviewed the data and coding for women reporting serious morbidity. Data coding and review were blind to treatment allocation.

### Outcome measures

The main outcome for women in the follow-up study was prespecified as a composite measure of death or serious morbidity potentially related to pre-eclampsia.^13^ This included women who died either before or after discharge from the hospital and those who survived but had one or more of renal problems, stroke and severe hypertension at follow-up.

Hypertension and other health problems reported by the women were secondary outcomes as were the reported use of health service resources and use of prescribed drugs.

### Sample size

Power of the study was estimated based on outcome for the children.^12^,^13^ Nevertheless, we estimated that 2800–3350 women would be eligible for follow-up and that approximately 20% would not be contacted. After adjusting for this expected loss to follow-up, it was anticipated that data would be available for 2405–2850 women, 2095–2485 randomised before delivery and 310–365 randomised after delivery.

### Statistical analyses

Analyses were based on the groups to which the women had been allocated at trial entry. Centres able to contact less than 20% of included families were excluded. For the main outcome, statistical significance was taken as the 5% level. Where appropriate, results are presented as relative risk (RR) with 95% confidence intervals. Women who died were excluded from the analysis of other health problems and use of health service resources.

Planned subgroup analyses^13^ were based on severity of the woman’s pre-eclampsia at trial entry (severe, moderate or mild), whether randomised before or after delivery and perinatal mortality (PNM) in the country (high, medium or low).^15^ Severe pre-eclampsia was as defined in the original trial protocol.^8^ The definitions of moderate and mild pre-eclampsia^13^ were agreed before the analysis of outcome at discharge from the hospital. These two groups were previously reported as ‘nonsevere’ pre-eclampsia.^8^ PNM for each country was grouped, as in our previous report,^8^ a high PNM, more than 40 deaths per 1000 births; moderate, 20–40 deaths per 1000 births and low, less than 20 deaths per 1000 births.^15^

## Results

Overall, 125 centres in 19 countries in Africa, Asia, USA, Australia and Europe participated in the follow-up. The coordinating centre in Oxford traced families from the 67 UK centres and provided support to local collaborators who traced all other families. Data collection closed on 31 December 2003. Excluded were 466 women discharged from hospital without a surviving child, 26 who opted out and 109 from a centre that contacted less than 20% of families. Of the women discharged without a surviving child, 36% had severe pre-eclampsia at trial entry (58 of 169 versus 109 of 297), 52% had moderate pre-eclampsia (84 versus 156) and 13% had mild pre-eclampsia (27 versus 32). Included in the follow-up study were 4782 women (magnesium sulphate, *n* = 2374 and placebo, *n* = 2408) (Figure 1), 16 (0.3%) who died before discharge from the hospital and 4766 who were alive at discharge from the hospital.

Women included in the follow-up were similar to those in the trial overall. The only substantive differences were that a higher proportion of women included in follow-up came from low or middle PNM countries (65% versus 44% in trial overall); so more of them received the intravenous maintenance regimen for magnesium sulphate (67% versus 46%).^8^ In addition, fewer women included in the follow-up were recruited at or before 33 completed weeks than those in the trial overall (19% versus 27%).

### Completeness of data

Data for 3375 women (71% with a response) were available for the analysis, of whom 2809 were randomised before delivery (Figure 1). For 143 women in the UK, information was from the GP only. In the UK, 98% of women were contacted. Outside the UK, 11 centres contacted all included women and 36 contacted more than half of the included women. Data were available for 49% of women in high PNM countries (807 of 1651), 74% in middle PNM countries (1365 of 1857) and 95% in low PNM countries (1203 of 1272) (Table 1).

**Table 1:** Characteristics at trial entry, exposure to magnesium sulphate and outcome at discharge from hospital for women included in follow-up and those who responded

	Women included in follow-up		Women with response	
		
	MgSO_4_, *n* = 2374	Placebo, *n* = 2408	MgSO_4_, *n* = 1650	Placebo, *n* = 1725
**Characteristics of the women**				
Singleton pregnancy	2282 (96)	2322 (96)	1589 (96)	1662 (94)
Pre-eclampsia				
Severe	528 (22)	564 (23)	385 (23)	408 (24)
Moderate	1071 (45)	1050 (44)	699 (42)	733 (43)
Mild	775 (33)	794 (33)	566 (34)	584 (34)
Prior anticonvulsant	139 (6)	164 (7)	89 (5)	105 (6)
≤33 completed weeks*	370 (19)	369 (18)	239 (17)	238 (17)
Postpartum	384 (16)	412 (17)	268 (16)	298 (17)
**Characteristics of the centre**				
Intravenous maintenance regimen	1607 (68)	1613 (67)	1250 (76)	1275 (74)
High PNM country	817 (34)	834 (35)	386 (23)	421 (24)
Middle PNM country	927 (39)	932 (39)	671 (41)	694 (40)
Low PNM country	630 (27)	642 (27)	593 (36)	610 (35)
**After trial entry**				
No exposure to MgSO_4_	34 (2)	2330 (97)	29 (2)	1663 (96)
**Outcome at discharge from the hospital**				
Severe morbidity in hospital**	92 (4)	105 (4)	62 (4)	72 (4)
Admission to high care	1227 (52)	1237 (51)	857 (52)	882 (51)
Caesarean section*	1091 (55)	1023 (51)	776 (56)	728 (51)

Data are *n* (%).

* Women randomised before delivery only: included women in MgSO_4_ group, *n* = 1990 and in placebo group, *n* = 1996; traced women in MgSO_4_ group, *n* = 1382 and in placebo group, *n* = 1427.

** Includes eclampsia.

### Outcome for the women

Outcome at discharge from hospital was similar for women included in the follow-up and for those with a response (Table 1). Five percent of surviving women responded to the questionnaire within 12 months of delivery, 32% responded 13–24 months after delivery, 39% responded 25–36 months after delivery, 16% responded 37–48 months after delivery and 4% responded more than 48 months after delivery. Timing of response was similar in the two treatment groups. For 5% of women in both groups, data were only available from the UK GP. Twenty-two percent of women in both groups had had another pregnancy since the Magpie birth. At the time they completed the questionnaire, 85 women allocated magnesium sulphate were pregnant, as were 95 of those allocated placebo. There were 62 women (1.8%) whose child had died after discharge from the hospital (31 in each group).

**Figure 2:**
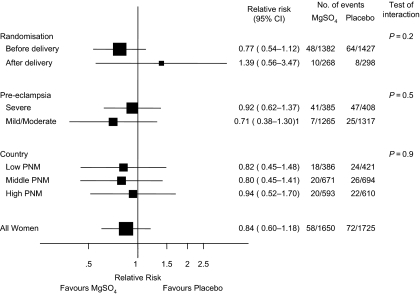
Death or serious morbidity related to pre-eclampsia at follow-up: prespecified subgroups.

Fifty-eight of the 1650 (3.5%) women allocated magnesium sulphate died or had serious morbidity potentially related to pre-eclampsia compared with 72 of the 1725 (4.2%) women allocated placebo (RR 0.84, 95% CI 0.60–1.18) (Figure 2). Almost one-quarter of the women reported having high blood pressure since their Magpie pregnancy (Table 2). Of the women who did not report chronic hypertension, 219 of 1604 (14%) allocated magnesium sulphate had taken one or more antihypertensive drugs for at least 2 weeks since their Magpie pregnancy as had 250 of 1664 (15%) women allocated placebo, and 106 of 1604 (7%) allocated magnesium sulphate were taking antihypertensive(s) at the time of assessment as were 127 of 1664 (8%) women allocated placebo.

**Table 2:** Death, serious morbidity potentially related to pre-eclampsia and hypertension for all women with response

	MgSO_4_, *n* = 1650	Placebo, *n* = 1725
**Death**	18 (1.1)	17 (1.0)
Died before discharge from the hospital	6	10
Died after discharge from the hospital	12	7
Serious morbidity potentially related to pre-eclampsia*	40 (2.4)	55 (3.2)
Stroke	—	3
Serious renal problems	5	8
Severe hypertension**	35	47
**Died or had serious morbidity potentially related to pre-eclampsia**	58 (3.5)	72 (4.2)
**Hypertension**		
Chronic hypertension	46 (3)	61 (4)
Reported having high BP before Magpie pregnancy***	57 (4)	62 (3)
Reported having high BP since Magpie pregnancy***	325 (20)	369 (21)
Taken antihypertensive drug since Magpie pregnancy****	253 (15)	297 (17)
≥2 drugs since Magpie pregnancy****	64	88
**Taking antihypertensive drug ‘now’**	136 (8)	167 (10)
≥2 drugs ‘now’	28	37

Data are *n* (%).

BP, blood pressure.

* Some women had more than one health problem.

** Defined as hypertension and ≥2 antihypertensive drugs reported on the questionnaire.

*** Excludes women with chronic hypertension, those with hypertension only on oral contraceptives and those with hypertension only during another pregnancy.

**** Excludes any antihypertensive drug taken within the first 6 weeks after birth.

Two-thirds of the surviving women in both groups reported at least one health problem (Table 3). The only outcome for which the difference between the groups achieved statistical significance was gynaecological problems, for which the risk was higher in the magnesium group (RR 1.59, 95% CI 1.17–2.16). One hundred and fifty-four women (4.6%) reported having psychosis or depression, requiring treatment, of these 147 (95%) were from countries with low PNM. In total, 195 women (5.8%) reported one or more mental health problems. At least one mental health problem was reported by 184 of 1200 (15.3%) surviving women recruited in low PNM countries, by 8 of 1352 (0.6%) surviving women recruited in middle PNM countries and by 3 of 788 (0.4%) surviving women recruited in high PNM countries. There were no clear differences between the groups in the consumption of health services (Table 4). Health problems and use of health service resources were similar for women randomised before and for those randomised after delivery (data not shown).

**Table 3:** Other reported health problems for surviving women with response

	MgSO_4_, *n* = 1632	Placebo, *n* = 1708
Gynaecological problems*, *n* (%)	99 (6)	65 (4)
Menstrual disorder	46	31
Ovarian cyst or polycystic ovaries	3	3
Other or not specified	63	40
Minor ailment only**, *n* (%)	266 (16)	268 (16)
Other health problems*, *n* (%)	343 (21)	333 (19)
Psychosis or depression requiring treatment***	71	83
Asthma or other respiratory problem	40	32
Serious infection	22	31
Mental health problem not requiring treatment***	30	27
Gall bladder disease	12	19
Urinary tract infection or calculi	10	19
Liver problem	14	18
Diabetes	15	16
Thyroid disease	24	14
Cohn’s disease, ulcerative colitis, irritable bowel syndrome	15	14
Trauma	6	14
Cardiac problem (includes CHD and RHD)	6	11
Cancer	8	9
Epilepsy	9	7
Thromboembolic disease	5	5
Minor ailment plus other morbidity	189	188
Other or not specified	15	7
At least one health problem****, *n* (%)	1015 (62)	1059 (62)

CHD, congential heart disease; RHD, rheumatic heart disease.

* Some women had more than one health problem.

** Minor ailments included complaints such as colds, coughs, flu, back problems, anaemia, piles and breast pains.

*** Treatment included drugs and/or other forms of therapy.

**** One or more of hypertension, gynaecological problem, morbidity potentially related to pre-eclampsia, other health problem, minor ailment, visit to a clinic (excludes family planning or postnatal care) or admission to hospital (excludes for another pregnancy).

**Table 4:** Use of health service resources and drugs other than antihypertensives for surviving women with response

	MgSO_4_, *n* = 1632	Placebo, *n* = 1708
**Use of health service resources since delivery, *n* (%)**		
Attended clinic	527 (32)	583 (34)
Hospital admission	158 (10)	168 (10)
Reason(s) for hospital admission*		
Surgery	37	31
Hypertension	17	33
Infection/HIV	9	16
Gastrointestinal problem	16	15
Psychiatric problem	4	5
Renal problem	4	4
Cardiac problem	1	4
Gynaecological problem	17	3
Stroke	—	1
Thromboembolic disease	4	1
Minor ailments	18	23
Other**	19	21
Drugs other than antihypertensives taken for >2 weeks***, *n* (%)	269 (16)	233 (14)
Antidepressant/sedative	59	64
Analgesia/anti-inflammatory	37	34
Antibiotic/antifungal	22	25
Gastrointestinal treatment	18	17
Asthma treatment	14	13
Diabetes treatment	7	11
Thyroid treatment	16	9
Steroid	8	3
Drug name not known	26	21
Other	33	21

* Excludes admission related to another pregnancy.

** Includes investigation, sterilisation, cancer, diabetes, epilepsy, arthritis, trauma, thyroid, serious liver problems and ear/hearing problems.

*** Excludes nutritional supplements and contraception.

Overall, two-thirds of the surviving women contacted (2136 of 3340) were interviewed alongside their child‘s assessment, 1513 were interviewed within a week of responding to the questionnaire, 222 within 2–4 weeks, and 382 after 4 weeks. Women who were interviewed were more likely to have reported that they had high blood pressure on the questionnaire (386 of 1046 [37%] versus 447 of 1072 [42%]) than women with a response overall (Table 2). Blood pressure was measured during the interview for 1781 women, of whom 8% were taking antihypertensive drugs at the time of the interview (66 of 865 versus 88 of 916). Seventeen percent of women had either systolic blood pressure ≥140 mmHg or diastolic blood pressure ≥90 mmHg (134 of 865 versus 178 of 916), of whom one-third were taking an antihypertensive at the time of the interview (39 versus 56). The proportion of women with raised blood pressure taking an antihypertensive drug was similar across the three categories of country.

## Discussion

The use of magnesium sulphate for women with pre-eclampsia was associated with a 16% reduction in the risk of death or serious morbidity potentially related to pre-eclampsia 2–3 years later. The confidence intervals suggest that the true effect could be anything from a 40% reduction to an 18% increase. Despite having prespecified a composite outcome, our study had insufficient power to show a clear difference. To illustrate the sort of sample size needed, for the placebo group event rate of 4.2%, 26 000 women would have been required to show a 16% difference in death or serious morbidity potentially related to pre-eclampsia (*α*0.05, power 80%). Nevertheless, it is reassuring that the point estimate consistently favours women allocated magnesium sulphate (Figure 2). These data also provide reassurance that the use of magnesium sulphate to reduce the risk of eclampsia is not associated with unexpected adverse events for women in the medium term. This reassurance is in addition to the data we have already presented, showing no clear difference in the risk of death or neurosensory disability at the age of 18 months for children randomised while *in utero*.^12^

Although 50 centres were unable to participate in the follow-up, this could not have introduced bias into the assessment of outcome, as randomisation had been stratified by the centre. As discussed in our accompanying article,^12^ for the 125 centres that did participate in the follow-up, it is implausible that there was substantive bias in the selection of families, as this process was conducted blind to the treatment allocation. Bias in the assessment of outcome is similarly implausible as this was also conducted blind to the treatment allocation.

Inevitably, it was centres in low PNM countries that were most likely to participate in the follow-up and were most successful at contacting the women. Nevertheless, one-quarter of women with a response were from high PNM countries.

Although pre-eclampsia appears to be associated with cardiovascular morbidity and mortality in later life,^6^,^7^ it remains unclear whether this association is causal. Nevertheless, interventions to improve outcome following pre-eclampsia might also improve longer term outcome for the women. In this follow-up study, although the numbers were small and there was no clear difference between the two groups at the follow-up assessment, fewer women allocated magnesium sulphate had a stroke (zero versus three, serious renal problems (five versus eight) or severe hypertension (35 versus 47).

The Magpie Trial recruited a cohort of women with pre-eclampsia; 2–3 years later, one in a hundred of these women had died and a further three in a hundred had serious morbidity potentially related to pre-eclampsia. About one-quarter of the women reported having persistent high blood pressure after the pregnancy in which they were recruited to the trial, and one in ten were taking at least one antihypertensive drug at the time of assessment. Although our study does not provide data for a comparable group of women without pre-eclampsia, this morbidity and mortality does seem higher than might be expected following uncomplicated pregnancy. For example, comparing outcome in the placebo group for women with severe pre-eclampsia at trial entry with that for those who did not have severe pre-eclampsia, there was a six-fold difference in death or serious morbidity potentially related to pre-eclampsia: 12.2% (47 of 385) for women with severe pre-eclampsia compared with 1.9% (25 of 1317) for those without. We did not attempt to assess women who left hospital without a surviving child. These women were more likely to have had severe pre-eclampsia at trial entry and so might be expected to have higher levels of morbidity and mortality at follow up. Therefore, our data may still have underestimated the true risk of death or serious morbidity related to pre-eclampsia.

Only one-third of surviving women did not report any health problems. Information reported here was provided by the women in response to questions about what problems had led them to consult a doctor, attend a clinic or be admitted to hospital. Hence, our data may underestimate the true morbidity as women may have had problems but, for a variety of reasons, not attended the health services. This is particularly likely to be the case for women in the high and middle PNM countries. For example, reported mortality was highest in high PNM countries (1.6% for high PNM countries, 1.0% for middle PNM countries and 0.4% for low PNM countries), while serious morbidity potentially related to pre-eclampsia was reported to be highest in low PNM countries (1.9% for high PNM countries, 2.4% for middle PNM countries and 4.8% for low PNM countries). Gynaecological problems appeared to be more common among women allocated magnesium sulphate. This was an unexpected finding, and the most likely explanation is that this is due to chance.

Depression is common following childbirth. In developed countries, the prevalence of postnatal depression is around 13%.^16^ Within developing countries, depression after childbirth may be even more prevalent, with levels of 30% or above reported. 17,18 These estimates are largely based on prospective studies that used self-completed questionnaires such as the Edinburgh Postnatal Depression Scale and the Beck Depression Inventory and/or interviews. In contrast, we asked women to report in their own words problems they had experienced. We therefore expected that some women with postpartum depression or other mood disorders would not report this, purely because their symptoms had not been recognised or given a medical label. The 6% of women who reported having at least one mental health problem almost certainly represent substantial underascertainment. In particular, the low level reported by women in middle and high PNM countries, less than 1% compared with 15% in low PNM countries, should not be taken as a measure of the true psychiatric morbidity. Lack of awareness and stigma may well have contributed to underreporting. In addition, women at greatest risk of postnatal depression may have been more likely to opt not to respond to our questionnaire,^19^ and one group of women at high risk for depression, those discharged without a surviving child, were excluded from our study.

Our study was unique in contacting and assessing outcome for women and their children after pregnancy complicated by pre-eclampsia. The wide range of countries in which women were contacted also means that with the limitations outlined above, our results are generalisable to women in low, middle and high PNM countries.

## Conclusions

The reduction in the risk of eclampsia following prophylaxis with magnesium sulphate was not associated with an excess of death or disability for the women after 2 years.

## Magpie trial follow up study collaborative group

### Central coordination and coordinating team for UK

Lelia Duley (clinical coordinator), Barbara Farrell (trial manager), Nina Armstrong (follow-up study coordinator), Patsy Spark (trial programmer), Barbara Roberts (trial secretary), Rebecca Smyth (research midwife), Mary Tivnan (data manager), Anna Laws (research assistant), Noelene Corfield (data assistant), Annette Salter (data assistant) and Lisa Thorn (data assistant).

### Statistical support

Douglas Altman and Ly-Mee Yu of Centre for Statistics in Medicine and Patsy Spark of Central coordinating team.

### Coordinating team for Argentina

Edgardo Abalos (clinical coordinator), Berenise Carroli (coordinator), Lucrecia Dellepiane (field worker), Marina Duarte (paediatrician), Hebe Fernandez (field worker) and Daniel Giordano (programmer).

### Management group

Douglas Altman, Nina Armstrong, Mike Clarke, Lelia Duley, Barbara Farrell (chair), Alastair Gray, Edmund Hey, James Neilson, Judit Simon, Patsy Spark, Rebecca Smyth and Ly-Mee Yu.

### Steering committee

Douglas Altman, Rory Collins, Lelia Duley (observer), Barbara Farrell (observer), Anna Karaoglou (observer), Richard Lilford, Jack Moodley, James Neilson (observer), Stephen Robson, Ian Roberts, Peter Rubin (chair), James Thornton, Sara Twaddle, José Villar (observer), Isabel Walker and Christopher Watkins (observer).

### Writing committee

Edgardo Abalos, Douglas Altman, Nina Armstrong, Lex Doyle, Lelia Duley (chair), Barbara Farrell, Edmund Hey, Jack Moodley, James Neilson, Patsy Spark, Rebecca Smyth and Ly-Mee Yu.

### Collaborators by country

#### Albania

Maternity Hospital No. 2: A Bimbashi, E Demalia, O Gliozheni and A Shpata.

#### Argentina

Hospital Carlos A Durand: A Karolinski, M Lamas, M Pesaresi and V Wainer; Hospital Del Centenario: W Barbato; Hospital Eva Perón: M Paciocco; Hospital Interzonal Dr José Penna: M Bertin, E Boiza, J Castaldi and Y Partida; Hospital J B Iturraspe and Hospital Jose Maria Cullen: C Arias, M Farri and G Kerz; Hospital Juan R Vidal: J Aguirre, M de Sagastizábal, R Falcone and E Morales; Hospital Maternidad Martin: G Carroli; Hospital Nacional Prof. Alejandro Posadas: S Krupitzky, S López, M Palermo and D Montes Varela; Hospital Provincial Santa Fé: H Delprato; Hospital Regional ‘Dr Ramón Carrillo’: H Camusso and M Curioni; Hospital Roque Saénz Peña: E Ludmer; Luis Carlos Lagomaggiore: R Brandi, R Martin, W Mesas and R Taralli; Maternidad Provincial Salta (Nuevo del Milagro): M Lezaola and M Morosini; Materno Infantil ‘Ramón Sardá’: E Andina, L Bernal, M Estiú and E Ulens; Materno Provincial Córdoba: B Ortiz de Speranza and A Peyrano; Zonal de Agudos ‘Héroes de Malvinas’: M Damiano and C Saumench.

#### Australia

Royal Brisbane & Women’s Hospital Perinatal Research Centre: J Horn, M Pritchard, V Smith-Orr and M Wilson; The Townsville Hospital: A Lawrence and D Watson; Women‘s and Children’s Hospital: C Crowther and J Paynter.

#### Bangladesh

Bangabandhu Sheikh Mujib Medical University: Ashrafunnessa, M Mannan, M Shahidullah and L Shamsuddin.

#### Brazil

Hospital das Clinicas Da UFPE: C Barros Santos, S Freire and E Melo.

#### Colombia

Fundaciòn Clínica Valle del Lili: E Cobo and M Jaramillo; Universidad Nacional de Colombia, Hospital Materno Infantil de Bogotá: C Cardozo, N Fandiño, H Gaitán and L Montaño; Hospital Universitario de San Ignacio: J Lozano and M Rojas.

#### Cuba

Docente Gineco-Obstétrico ‘Eusebio Hernández’: A Breto Garcíá, A Fuentes Ramirez, R Garcíá Mírás and S Sampera; Hospital Gineco-Obstétrico ‘América Arias’: U Farnot, E Gómez, G Rojas and R Valdés.

#### Egypt

Assiut University Hospital: H Abd El-Kreem, T Al-Hussaini and E Hammad.

#### Ghana

Komfo Anokye Teaching Hospital: K Danso, E Kwapong and F Ofosu-Barko.

#### India

Christian Medical College Hospital: M Padmini Jasper, A Peedicayil and A Regi; J N Medical College AMU: R Sharma; King Edward VII Memorial Hospital: A Chauhan, V Raut and R Udani; Maulana Azad Medical College and Loknayak Hospital: S Batra, A. Muthal-Rathore, S Ramji and V Zutshi; SAT Hospital, Medical College Trivandrum: S Balakrishnan, E Eapen and G Koshy; Shree Maharani Shantadevi Hospital: B Ambardar, P Vadakkepat and D Vaidya.

#### Malawi

Queen Elizabeth Central Hospital: V Lema, Y Rijken and E Tadesse.

#### Nigeria

Centre for Research in Reproductive Health (coordination of hospitals in Sagamu region): O Dada and A Sofekun; Nigerian Security Printing and Minting Staff Hospital: C Ohiaeri; Olabisi Onabanjo University Teaching Hospital: T Runsewe-Abiodun; University College Hospital: I Adewole, A Adeyemo, B Brown and R Oladokun; University of Port Harcourt: O Adewale, N Inimgba, C John and R Ogu; Usmanu Danfodiyo University Teaching Hospital: B Ekele, A Isah and B Onankpa.

#### Pakistan

Jinnah Post Graduate Medical Centre: R Jamelle and D Junejo; Lady Reading Hospital: N Faiz, F Gul and A Sherin; Maternal and Child Health Centre: K Bangash, G Mahmud, K Masud and N Tasneem.

#### Sierra Leone

Princess Christian Maternity Hospital: S Gassama and A Soyei.

#### Singapore

KK Women’s and Children’s Hospital: P Agarwal and V Rajadurai.

#### South Africa

Chris Hani Baragwanath Hospital: N Pirani; University of Pretoria (Kalafong Hospital and Pretoria Academic Hospital): S Delport, P Macdonald, R Mokhondo, R Pattinson and M Zondo; King Edward VIII Hospital: M Adhikari, N Mnguni and J Moodley; Tygerberg Hospital: M Carstens, G Kirsten, W Steyn and J van Zyl.

#### USA

OHSU Hospital: A Helwig, S-L Jacobson and R Panosche.

#### Zimbabwe

Harare Hospital: E Hammond and L Masanganise.
